# Presuppositions, cost–benefit, collaboration, and competency impacts palliative care referral in paediatric oncology: a qualitative study

**DOI:** 10.1186/s12904-022-01105-0

**Published:** 2022-12-02

**Authors:** Naveen Salins, Sean Hughes, Nancy Preston

**Affiliations:** 1 Department of Palliative Medicine and Supportive Care, Kasturba Medical College, Manipal, India; 2 Manipal Academy of Higher Education, Manipal, Karnataka 576104 India; 3 Division of Health Research, Health Innovation One, Lancaster University, Sir John Fisher Drive, Lancaster, LA1 4AT UK

**Keywords:** Oncologists, Haematologists, Child, Cancer, Referral, Palliative care

## Abstract

**Background:**

Although a significant proportion of children with cancer need palliative care, few are referred or referred late, with oncologists and haematologists gatekeeping the referral process. We aimed to explore the facilitators and barriers to palliative care referral.

**Methods:**

Twenty-two paediatric oncologists and haematologists were purposively recruited and interviewed. Data were analysed using reflexive thematic analysis. Findings were interpreted using the critical realist paradigm.

**Results:**

Four themes were generated. 1) Oncologists expressed concern about the competency of palliative care teams. Palliative care often symbolised therapeutic failure and abandonment, which hindered referral. Trustworthy palliative care providers had clinical competence, benevolence, and knowledge of oncology and paediatrics. 2) Making a palliative care referral was associated with stigma, navigating illness-related factors, negative family attitudes and limited resources, impeding palliative care referral. 3) There were benefits to palliative care referral, including symptom management and psychosocial support for patients. However, some could see interactions with the palliative care team as interference hindering future referrals. 4) Suggested strategies for developing an integrated palliative care model include evident collaboration between oncology and palliative care, early referral, rebranding palliative care as symptom control and an accessible, knowledgeable, and proactive palliative care team.

**Conclusion:**

Presuppositions about palliative care, the task of making a referral, and its cost-benefits influenced referral behaviour. Early association with an efficient rebranded palliative care team might enhance integration.

**Supplementary Information:**

The online version contains supplementary material available at 10.1186/s12904-022-01105-0.

## Introduction

Worldwide, every year around 300,000 children are diagnosed with cancer [1], of whom 90% live in low and middle-income countries, constituting 84% of the global burden of childhood cancers [2]. A significant disparity in cure rate and mortality between high and low-income countries makes palliative care highly relevant in a paediatric oncology setting [3, 4]. Empirical studies have shown that palliative care referral in a paediatric oncology setting improves the quality of life [5–8], pain and physical symptoms [6, 9–17], emotional support to children and their families [10, 15, 18–21], communication between families and health care providers [11, 15, 16, 19–26], advance care planning and end-of-life care support [5, 12–16, 18, 20, 26–32].

Although many children with cancer need palliative care, 65.6% of countries have no known paediatric palliative care activity [33, 34]. Diminished access is primarily due to limited public and health care providers’ understanding of the role of palliative care [35]. Furthermore, children in low and low-middle income countries are less likely to access palliative care due to sparce number of palliative care services [35]. Globally children with cancer are infrequently referred to palliative care and late in the illness trajectory [15, 36–40]. Children with haematological malignancies are referred even less [41–45], and most receive some form of chemotherapy in their last days [39, 40, 43, 44]. Cure potential, complex course and complications needing intense medical attention often hindered palliative care referral in paediatric blood cancer setting [46]. Non-referral and delayed referral often leads to invasive medical interventions at the end of life [40, 42] and increased in-hospital deaths [39, 40, 42, 47]. Oncologists and haematologists act as gatekeepers, and their views about palliative care referral can facilitate or hinder referral to palliative care [48, 49]. Moreover, they have the discretionary authority to make treatment and referral decisions for their patients [50]. We believe that clinical research should have an emancipatory goal where the research inquiry intertwined with a social action agenda has the potential to change the lives of people and the society in which we live and work [51]. Critical realism is a philosophical basis for transformative research [52]. Therefore, critical realist paradigm informed the conduct of this research and interpretation of study findings.

To ensure enhanced integration of paediatric oncology and paediatric palliative care, there is a need to explore the facilitators and barriers for referral internationally in various practice settings. Except for the two studies [53, 54] conducted in high-income countries, all other studies examined oncologists' views in the adult setting. This was the first study exploring paediatric oncologists’ perspectives on palliative care referral in a low-middle-income country (LMIC) setting. In this study we explored the perspectives of paediatric oncologists and haematologists on what helps or hinders the palliative care referral of a child with cancer.

## Methods

### Aim and study setting

This study aimed to explore the views of paediatric oncologists and haematologists on what facilitates or hinders the referral of a child with advanced cancer to palliative care. The study was conducted in 13 cancer centres across India. The sites for qualitative interviews were selected based on three essential criteria: a) sites where paediatric oncology and haematology services were offered, b) availability of oncologists and haematologists managing children with cancer, and c) availability of palliative care services to which children could be referred to. Most cancer centres participating in this study were in urban cities, except for two in semi-urban areas. The research question was *What are the views of oncologists and haematologists of what facilitates or hinders referral of a child with advanced cancer to palliative care?*

N.B. Henceforth Oncologist(s) means Oncologist(s) and Haematologist(s).

### Participant recruitment

The inclusion and exclusion criteria of the research participants are provided in Table 1. Eligible paediatric oncologists were purposively identified and invited to participate. Purposive sampling enabled identifying participants with experience of the phenomenon studied. Moreover, adherence to the eligibility criteria during selection enabled homogeneity of the participant’s perspectives [55]. Reflexive thematic analysis is an approach to qualitative research which relies on the researcher’s subjectivity, depth of engagement and reflexive interpretation [56]. The purpose was to develop a rich, complex, and multi-faceted story of the phenomenon that had been explored. Therefore, as described in other qualitative methodologies and methods, saturation as the generic quality marker for sample size determination was not adopted [57, 58].

**Table 1:** Selection criteria of study participants

	**Inclusion**	**Exclusion**
Participants	Paediatric oncologists and paediatric haematologists Adult medical oncologists and adult haematologists if 25% of their clinical practice involved treating children with either solid or haematological malignancy Consultant level practice	Trainee oncologists and haematologists Radiation and surgical oncologists
Setting	Cancer hospitals or tertiary hospitals with cancer care services Have access to palliative care services	Private oncology and haematology consulting suites without inpatient facility situated outside a hospital setting Hospital where the researcher is working

### Qualitative interviews

After consent, research data were collected through individual, face-to-face, semi-structured qualitative interviews. When conducting and analysing the interviews, a critical realist perspective was used to formulate the semi-structured interview topic guide, which is provided as a supplementary file. The interview topic guide was developed from a systematic review conducted by the authors [59].

The critical realist paradigm focuses on the ontological depth of phenomena, knowing the multi-layered nature of social reality and the intertwined generative mechanisms within these layers, causing a social event [60]. The phenomenon of palliative care referral might have several generative mechanisms causing the event. Some are empirically observed, and some are deeper and hidden, restricting understanding of the ontology [61]. However, the deeper and hidden mechanisms known as actual and real mechanisms in critical realist terms are the preconditions for an event to happen [62]. These generative mechanisms may be layered as biological, physical, psychological, social, and organisational, and a complex interplay between these layers could influence the event [62].

Critical realist interviews are theory-driven [60]. The study researchers have expertise in the subject, understand the context, and have their views about the generative mechanisms leading to palliative care referral [63]. This facilitated exploration of the explanatory mechanisms discussed by the participants that focussed on motivations, reasoning, attitudes, choices and decision-making [63]. Therefore, the researcher’s theory and participant’s view facilitated the co-creation of knowledge during research interviews and analysis. Being reflexive during the interviews enabled researchers to clarify and elaborate upon participants’ views, which facilitated a collective understanding of participants’ perspectives and experiences [64]. All interview transcripts were uploaded and coded using NVivo software version 12.6.0 for Mac. The interview transcripts and a sample coded interview transcript are provided as supplementary files.

### Data analysis

Research data were analysed using the systematic six-step approach outlined in Braun and Clarke’s Reflexive Thematic Analysis method [56]. This reflexive approach uses the researcher’s subjectivity as a resource during data analysis [56]. In a reflexive approach, themes are meaning-based patterns, conceptualised and analysed by the researcher and not merely a superficial summary of the data. It involves the significant critical engagement of the researcher with the dataset, where the researcher is actively interpreting the data through the lens of their scholarly knowledge, socio-cultural view, ideology, and theoretical suppositions [56]. The personal beliefs and presuppositions that may skew data collection, analysis and interpretation of study findings were logged in a reflexive journal [65, 66]. This helped the researchers to have an open mind during interviews and data analysis. The data analysis process was checked using a 15-point checklist of criteria for good thematic analysis consistent with our reflexive approach [67].

### Ethics approval

The study was approved by the Indian (Kasturba Medical College/Kasturba Hospital Institutional Ethics Committee 292/2018) and United Kingdom (Lancaster University- Faculty of Health and Medicine Research Ethics Committee 17,089).

## Results

Twenty-two eligible paediatric oncologists were purposively recruited from 13 tertiary cancer centres across seven cities in India. Twenty participants practised paediatric solid tumour oncology and paediatric haemato-oncology, and two practised only paediatric haemato-oncology. Fifteen participants were men, and seven were women. The participant’s experience as a specialist ranged from 1 to 19 years (median 6 years). Among the participants, ten completed their specialist paediatric oncology training in India, nine in the UK and one each from Australia, Singapore and the USA. A detailed description of the participants can be found in Table 2.

**Table 2:** Description of the research participants

Participant	Gender	Area of Specialisation		Oncology Training Site	Number of years as a consultant in India at the time of interview
		Paediatric Solid Tumours	Paediatric Haemato-Oncology		
P001	Male	Yes	Yes	UK	10
P002	Male	Yes	Yes	UK	12
P003	Female	Yes	Yes	India	3
P004	Male	Yes	Yes	India	1
P005	Male	Yes	Yes	India	2
P006	Male	Yes	Yes	UK	6
P007	Female	Yes	Yes	UK	19
P008	Male	Yes	Yes	UK	6
P009	Male	Yes	Yes	UK	10
P010	Male	Yes	Yes	UK	16
P011	Female	Yes	Yes	India	11
P012	Male	Yes	Yes	India	5
P013	Male	Yes	Yes	India	2
P014	Male	Yes	Yes	India	5
P015	Male	Yes	Yes	UK	6
P016	Female	No	Yes	India	1
P017	Male	Yes	Yes	Singapore	5
P018	Female	Yes	Yes	India	4
P019	Male	Yes	Yes	UK	8
P020	Male	Yes	Yes	Australia	7
P021	Female	Yes	Yes	USA	12
P022	Female	No	Yes	India	1

**Fig. 1:**
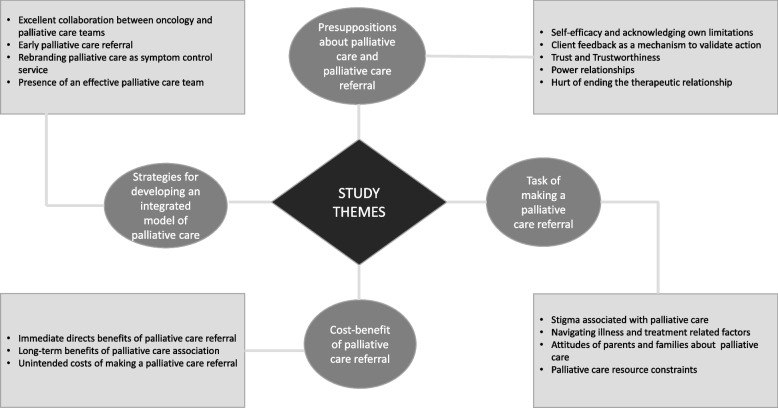
Thematic map of the research findings

Four themes were inductively generated from the study findings during data analysis. These were: 1) presuppositions about palliative care and palliative care referral, 2) the task of making a palliative care referral, 3) cost-benefits of making a palliative care referral, and 4) strategies for developing an integrated palliative care model in paediatric oncology. The study themes and subthemes are represented as a thematic map in Fig. 1. Illustrative quotes for these themes and their subthemes are provided in Tables 3, 4, 5 and 6.

**Table 3:** Presuppositions about palliative Care and palliative care referral

Subthemes	Participant Quotes
Self-efficacy and acknowledging own limitations	Symptoms like constipation, loose stools, some headaches, body pains. I think those are symptom care issues we as clinicians are trained to do that and we kind of manage it quite all right. (P 020) The pain management that we were giving in the end-stage was not good enough. The sedation that we were giving was not good enough, and I just felt that we were doing a very bad job. (P 016) The mother told me that it’s okay if he dies, because at least I have got somebody to listen to me and to my child [referring to the palliative care team]. So that was the first time I realised that we don’t really speak to them well. (P 011)
Client feedback as a mechanism to validate action	When somebody comes back, they themselves are in a very traumatic state to lose a child. And within a couple of weeks of the child’s funeral, to come back, seek us out and specifically mention what went well. It’s not just a general thanking, it was specifically about the care the boy received when he was dying. (P 007) Parents of some of the children who died came and thanked us which is very unusual. (P 001)
Trust and trustworthiness	You need to know whether they are going to do a good job of taking care because you have been taking care of them for such a long time. (P 016) So, faith in their competence is important (…) I would refer to somebody, but if that person is not very good then it is not helpful, it makes a huge difference. (P 007) They should have a paediatric perspective of dealing with care or symptom control (…) Dealing with a child is always different than dealing with an adult. (P 017) We are trained to look at things objectively (…) The response rate is 56% versus 63% (…) The overall survival is 90% versus 95%. Even if it’s not the same language used in palliative care, probably that clarity of thoughts is not present. (P 014) They were not wholeheartedly in it (…) I felt a lot of lack of empathy in there (…) the parents have come back and told me that it’s better that you handle this than them (…) I feel the compassion is a big thing in palliation. (P 003)
Power relationships	We are always a little more one level higher (…) A shared care concept has not come in (…) It’s more like I’m referring a patient to you, and you do what I want you to do and take care of them because of the seniority and also one is DM [highest subspecialist qualification in oncology], the other one is a DCH [basic diploma in paediatrics]. (P 011) They don’t deliver what I think they should deliver. Then I would not refer to them. (P 019)
Hurt of ending the therapeutic alliance	It is that kind of a feeling that you’re handing them over to somebody else, but you’re not sure whether they will take the same care of them and whether they will form the same bond with them. As I said they are like family. So, you know, it feels like you are handing over your part of your family to somebody and telling them, now you take care of them, but you always wonder whether they are doing a good job of it. (P 016)

(…) indicates part of the interview omitted by the authors for conciseness

**Table 4:** Task of making a palliative care referral

Subthemes	Participant Quotes
Stigma associated with palliative care	When you refer to the palliative team, some of the parents think that it is for end-of-life management (…) Parents think that some major issue is going on, and that’s why they have been referred to palliative care. (P 008) One thing is that family feels that you have sent to the doctor dealing with death. (P 006) Palliative care is associated with end-of-life care (…) If we have that thought process and we are not able to get over that thought process, how do we expect our patients and families to get over that thought process. (P 020) The family hears this word, palliative they say but you are trying to treat, cure my child why do you want me to engage with it [referring to palliative care]? (…) That means you’re telling me that my child is not really going to live. (P 009)
Navigating illness and treatment related factors	Child needs continued input in leukaemia, which is relapsed or refractory and continue to need blood support because there is going to be bleeding and they need blood all the time. So, these children we like to keep them under our cover. (P 007) You know, which way it is going to go. I usually refer to the palliative care team at that point before the child has got any symptoms. (P 001) If the curative option of treatment is going on, there will not be any referral to the palliative care. I don’t think that it will go well with the patient’s mind, but unless it is for symptom control. (P 006)
Attitudes of parents and families towards palliative care	What is really their role? They are not counsellors, they are not social workers, they are not doing psychological medicine. So, you know, what exactly is their role? that is what the family thinks and likes to know. (P 005) From the family perspective, they will think that the doctor has completely, you know, abandoned this patient by referring to other departments. (P 012) When a family is not ready, then there’s no point thrusting, they will agree for symptom management, but they want disease directed management as well. (P 010)
Palliative care resource constraints	It is difficult for patients with advanced malignancies to access palliative care or with complications to get admitted under palliative care. It is because of lack of inpatient beds and most of them had to be managed on outpatient basis, which again, becomes very difficult because titrating medicines and pain control on an outpatient basis becomes quite challenging. (P 005) Seeking and asking for referral is easy. If today every single deserving patient is sent to the department of palliative care of my hospital, they will be completely overwhelmed. I do not think that they have the people to handle that kind of a load. (P 014)

(…) indicates part of the interview omitted by the authors for conciseness

**Table 5:** Cost–benefit of palliative care referral

Subthemes	Participant Quotes
Immediate direct benefits of palliative care referral	We call the palliative team because they are much better at doing things like pain control than we are, so we utilise their expertise over there. We do that quite a lot especially in sarcomas when there is complex pain that we find difficult to control (P 007) It was so nice to see that difference because first three weeks, there was no interaction. Just nothing. Later she just opened up so much and family was happy. I am not saying happy would be the right word, but they were more accepting of the things, which I thought was really good (P 004) “Palliative team has made an effort to, you know, to be with them during the end of life, after the child has passed away, they have been with the parents. And they have made an environment which was very conducive for both parent and the child” (P 016) When we decided that the child is progressing on second line chemotherapy. They saw the palliative care team. Finally, the patient died at home, the parents actually phoned and said the child died, but you did everything, and palliative care did everything and thanked us. I think that is one of the things which was really the highlight as all these patients didn’t die in the hospital. They died at home. (P 001)
Long-term benefits of palliative care referral	It gives the chance of sharing the responsibility (…) Instead of you being the sole physician in charge, you get somebody else who will also be their doctor. It always helps to share responsibility, especially when things are not going well. (P 021) And when you struggle to take care of your normal patients, curative patients, or patient without symptoms, how would you spend time and resources for patients who need end of life care or symptom control. (P 017) I feel I have become more empathetic (…) I feel I have become much calmer as a person and It’s more of a positive effect. (P 003) I’m able to forecast and able to predict and able to pre-empt. So that is the skill that I’ve learned by engaging with palliative care physicians. (P 010)
Unintended cost of making a palliative care referral	I remain sceptical about the role of palliative during the curative setting because the worry is that Morphine is interfering with the ability to detect symptoms so that this abdominal pain could be a complication. And I don’t know if the child is getting worse, or there’s going to be respiratory depression and then the child will die because of that. (P 011) If you go and give information to family about a cancer and the cure rate and the relapse rate and something else, and the palliative care person team goes and completely paint a different picture, they may not be wrong. It’s just how you give the message. So, in a multi-disciplinary set up, the message has to be given consistently the same way. (P 019) If patients have to shell out more money because they have to see them, that becomes a deterrent. If that is built into the whole oncology costs, like if everything is paid for that includes palliative care team the patients don’t feel that they have to pay separately. (P 021)

(…) indicates part of the interview omitted by the authors for conciseness

**Table 6:** Strategies for developing an integrated model of palliative care

Subthemes	Participant Quotes
Excellent collaboration between oncology and palliative care teams	It is more of a personal relationship with the physician. So, they still want to see the oncologist, but if the palliative care team is there in the clinic and sitting with you and they recognise you as one of the physicians in the team, it will be easier for them to manage the patient. (P 015) While we are handling chemo, blood products, nutrition the palliative care team was taking care of her pain medicines, spending time with her, have a painting or drawing or just play some music and try to distract her. (P 003) I might be giving somebody a treatment which could save the child but at the same time it might not work, and it could go to a life extending or life ending situation. So, we bridge that gap with parallel planning where child is ready to receive both treatments and you choose what is the best. (P 009) If there is no proper communication between the two teams then it’s going to be a problem because even if I made a referral to the palliative care team unless we have discussed as to what is the purpose of making the referral? What are the goals of care? And what is the communication that has been made to the family? and vice versa expecting some kind of feedback from my colleague about the referral” (P 002)
Early palliative care referral	I find it very difficult to allow patients with the advanced cancer to die at home. That’s because, we have not involved palliative care right since the diagnosis (…) Rapport hasn’t developed. (P 013) Patients who need enucleation* or amputation, those undergoing chemotherapy… they are going through probably as much distress as a child who has relapsed at least for that period of time. (P 018) [*removal of the eye leaving behind the contents of the orbit intact] When I say that it’s not curative, they go to palliative care team. It’s almost like watershed, we don’t need watersheds. They can work in parallel. One team works takes the lead up to a point of time. The other team always remains in the background, helps whenever they need to, and if need to be, they become the primary team and the other team takes the backseat. (P009)
Rebranding palliative care as symptom control service	Not as a palliative care team, we counsel and tell them that this is the symptom control team which takes care of your pain, gives you supportive management, and will help you out. (P 018) When you say palliative care, I’m sure they’re [families] quite concerned and apprehensive about what’s going to happen to their kid. We tell them it is a symptom control program (…) Probably is an all-encompassing term and probably more comforting to the parents, and I would say for doctors as well. (P 022)
Presence of an effective palliative care team	They never waited for the referral from us, they used to do ward rounds and find out any child is having any kind of pain and or any kind of suffering they used to just point it out to us and suggest treatment and that way it was good. (P 013) When I pick the phone and I call them to discuss they were more than happy to discuss. We were able to discuss and come up with a consensus kind of plan. (P 002) It is not like you give a problem, like pain, you come and give Morphine, not that kind of palliative care, but actually assess, check, look at all the aspects. Okay, and then come up with a full plan rather than telling patient has pain give Morphine. (P 001)

(…) indicates part of the interview omitted by the authors for conciseness

### Presuppositions about palliative care and palliative care referral

Some participants felt they had the self-efficacy to address palliative care needs as they could manage pain and physical symptoms. Perceived self-efficacy is a person’s belief about their capabilities to produce a level of performance to accomplish a task successfully that meets the expectation of the self and others [68]. Participants thought that symptom management is an integral component of oncology care and that they have skills in counselling. However, the majority had a contradictory view of their ability to meet palliative care needs and acknowledged their limitations. They felt that their ability to assess and manage pain and physical symptoms was suboptimal and thought a specialist service should address these needs.

The participants believed that the feedback of parents and families referred to the palliative care team provided a valuable opinion of the quality of those services. Participants experienced families returning after the child’s death and thanking the oncologists for the end-of-life care provided. They felt it was usual for the families to thank the oncologists when the child was cured but unusual for them to thank the oncologists after the child’s death. Positive family feedback enhanced their confidence in the palliative care team, facilitating future referral.

The participants emphasised the need for a trusting relationship between oncology and the palliative care team and felt that a lack of trust is a barrier to referral. Participants perceived competence, training in oncology and paediatrics, assessment and management skills, and a benevolent approach made the palliative care providers trustworthy.

A minority of participants had concerns about the ability of palliative care providers to care for children with cancer as they lacked training in oncology and paediatrics, and a lack of clarity and objectivity in palliative care assessment and management made them less reliable. Undertreating and overzealous symptom management, a mechanistic approach, and a perceived lack of proactiveness and empathy by the palliative care providers hindered referrals.

A minority of participants thought that they had the power to make the palliative care referral. They felt that palliative care providers should agree to the line of clinical management proposed by the oncologists. They thought that the virtue of being an oncologist, expertise and qualifications, and years of professional experience conferred that power.

Some participants equated palliative care referral with a break in the therapeutic alliance. They believed that palliative care referral caused emotional hurt to oncologists and perceived it as a therapeutic failure and failing the child, which was attributed to a sudden shift in the treatment goals. Moreover, they believed that as oncologists, they are trained to cure and save and not give up. They compared referral to palliative care as handing over their family member and not seeing them again and breaching the bond established between them, which hindered a referral. The participant quotes linking the theme and subthemes are provided in Table 3.

### The task of making a palliative care referral

Participants expressed the presence of stigma among patients and families about palliative care due to a negative stereotyped association of palliative care with death, end of life and the terminal stage of illness. Participants sensed that families associated palliative care referral with a child not going to survive, a change in treatment intent, or stopping active treatment. They felt that families also perceived palliative care as a treatment offered just before death and palliative care physicians as doctors dealing with death. Participants believed families hesitated to pursue a line of management due to the stigma associated with its name. A few participants suggested that both oncologists and families would like to avoid the term palliative care. A few felt that oncologists perceive the term palliative care as a disadvantage, symbolic of a loss of hope and negative attributes.

Oncologists had to navigate complex permutations and combinations of illness-related factors that predisposed the patient for a referral. The course of illness, complications, stage of the disease, presence of symptoms, cure potential, the intent of treatment, prognosis and performance status of the patient were the predisposing factors influencing referral. Oncologists might find it challenging to navigate a complex set of illness-related factors underpinning the palliative care referral decision, which could make the referral task daunting.

The participants considered that parents and families lacked clarity about the role of palliative care, with parents and families having different perspectives about illness and a mismatch of goals and expectations. A lack of comprehension of the benefits of palliative care referral also hindered families from engaging with a palliative care team. Participants’ experience was of families being disinclined to attend meetings concerning palliative care referral. They presumed that discussing palliative care triggered a sense of abandonment, and families preferred the oncologists to continue the care instead of a new service provider caring for their child.

Most participants believed that patients and their families had restricted access to palliative care due to the limited availability of palliative care services, few inpatient beds and outpatient clinics, which hindered palliative care referral. Moreover, access to palliative care in rural and remote communities was almost non-existent, and families needed to travel long distances to access palliative care services. The participants thought that the capacity to provide care was limited due to fewer specialists, inadequate staffing to handle caseloads, limited space to provide palliative care and restricted access to opioids. The participant quotes linking the theme and subthemes are provided in Table 4.

### Cost–benefit of palliative care referral

Most participants appreciated the pain and symptom management as a benefit of a palliative care referral, believing that palliative care providers have exceptional skills in prescribing medications for symptom management. Furthermore, participants discussed several benefits of palliative care referral beyond the symptom management role. Participants thought that palliative care referral facilitated less intensive care and hospital resource utilisation and avoided potentially inappropriate medical interventions at the end of a child’s life. Moreover, engaging with the palliative care team helped parents and families better understand and accept the clinical situation, discuss prognosis, and participate in decision-making and future planning meetings. Palliative care referral facilitated access to end-of-life care, and end-of-life symptoms were well controlled. Families and caregivers were supported throughout the illness and beyond the child’s death. Participants felt that children referred to palliative care were more expressive and interactive and could communicate their intimate thoughts. Moreover, palliative care enabled normalising experiences for children as the team routinely involved them in play activities.

A few participants believed that a child who has completed all cancer-specific treatment should continue to live at home as enormous efforts are needed to transport the child back to the hospital. Moreover, parents and families found it stressful as they had to travel long distances to access care. Families preferred to stay at home and liked to be remotely supported from the hospital via the telephone or home visits from the palliative care team. The participants also preferred the children with no prospects of oncological treatment to be able to die at home. Dying at home enabled the child to be with the parents during the last moments and avoided unnecessary hospital-based interventions. Moreover, families also preferred their child to be at home during the terminal stages and thanked the oncologists for facilitating a home death.

Participants valued advantages of palliative care referral. By making a palliative care referral, they found a partner to share their responsibility of care. They felt that the palliative care team shouldered specific responsibilities like symptom management, family meetings, communication, counselling, and emotional support. The palliative care team also assisted oncologists in the medical management of the patients and in conducting minor procedures such as paracentesis. A few participants thought patients referred to palliative care had better treatment outcomes due to improved treatment compliance and reduced treatment abandonment rates. They also believed that making a palliative care referral can save oncologists' time on symptom assessment, management, and family communication. Moreover, participants felt that they had little time within their busy clinical practice to spend with children and their families, which could be compensated by the time provided by the palliative care team.

Some participants commented that the long-term association with the palliative care team also conferred several benefits towards their self-improvement as a doctor. Working with the palliative care team made them resilient and compassionate. It improved their confidence in managing children in the terminal stages and bettered their decision-making and prognostication skills. They also felt that working with the palliative care team enhanced their ability to handle emotions and counsel families.

Conversely, a few participants believed that palliative care referral during cancer treatment could cause interference in cancer management. Morphine prescribed by the palliative care team might mask cancer symptoms and lead to disease progression. It could mask the clinical signs of complications and worsen complications. Moreover, oncologists feared that morphine prescribed to children could cause addiction, respiratory depression, and hasten death. They disliked children receiving polypharmacy. They felt that the opportunity to resume chemotherapy is diminished if children are referred to palliative care.

Furthermore, a minority of participants felt that the palliative care team might provide conflicting information about the child’s clinical condition to the families, which could lead to a lack of congruence in communication and cause families to receive mixed messages about their child’s illness. They suggest this incongruence is due to palliative care providers having a different perspective of illness trajectory and treatment outcomes and poor inter-team communication. Moreover, participants disliked the palliative care provider’s perceived excessive discussion with the family about the child’s prognosis. The participant quotes linking the theme and subthemes are provided in Table 5.

### Strategies for developing an integrated model of palliative care

The participants perceived that limited awareness about the scope of palliative care hindered palliative care referral and that improved palliative care awareness and education of both consultants and trainee paediatric oncologists could enhance integration. Some participants believed that palliative care should be part of paediatric oncology training and curriculum, and paediatric oncology trainees should have palliative care rotations.

Participants emphasised the need for palliative care providers to be seen as part of the oncology team. A few opined that a physical presence during initial consultation was necessary even when there is a limited role for palliative care, and it was essential for families to recognise palliative care providers as part of the oncology team. They felt this could facilitate trust and bonding between families and the palliative care team and open communication channels. They liked palliative care providers participating in oncology clinics, ward rounds and family meetings, avoiding the need for a separate introduction to the palliative care team, a formal referral process and handover, and physical transfer of patients. Moreover, some felt that a referral-based service is less optimal than an integrated service and advocated for both teams to share the same work environment. They also thought that an experienced palliative care provider should participate during the initial consultation, as this facilitated better rapport-building between the palliative care team and families. Participants believed that both teams should understand and respect each other’s roles and that palliative care should be part of routine oncology management and should be provided to every patient.

Participants believed that cancer care and palliative care should be provided concurrently. The palliative care team has a role at diagnosis, during, and after treatment completion with the understanding that the tailored palliative care approach could complement the care provided by the oncologist. Moreover, oncologists felt that families perceive a concurrent care approach positively. Some participants suggested a parallel planning model where children will have both curative and palliative treatment planned. The child may receive either one or both based on individual needs.

Participants discussed the role of effective inter-team communication in facilitating a palliative care referral. Participants felt that communication must be transparent and bi-directional, and that clarity of communication facilitates referral. Inter-team communication should have a clear purpose that discusses patient management goals enabling both teams to arrive at a mutually agreed-upon plan. Communication need not always occur during the team meeting and can be an informal process or at a personal level. Participants liked palliative care providers giving feedback about the referrals made, helping both teams to be on the same page. Some participants preferred to be remotely involved and receive communications about the patients even when they were not directly involved in their care.

Participants felt the need for policy and referral guidelines for facilitating a palliative care referral. A few participants liked registering all children with cancer with palliative care and thought that nurses should be empowered to make palliative care referrals directly. Participants believed that referral criteria make it easy to refer, providing clarity and objectivity, which may help junior doctors and nurses make timely referrals. Some participants suggested creating an automatic referral trigger system based on pain scores, prognosis, and complications. The participant quotes linking the theme and subthemes are provided in Table 6.

## Discussion

In this study, the participants had mixed feelings about their self-efficacy to cater to palliative care needs. Specialist palliative care is delivered by trained specialist palliative care providers managing patients and families with complex symptoms, psychosocial needs and palliative care situations [69]. In contrast, the palliative approach or generalist palliative care refers to general practitioners or non-palliative care specialists with basic training in palliative care catering to simple palliative care needs [69]. In Canada, patients and families access generalist palliative care providers twice as much as they do specialists [70]. Generalist and specialist palliative care providers working together could create a more sustainable model as increasing demand for palliative care could outstrip the supply of specialist providers [71]. Effective collaboration between them could be achieved through proactive communication, role negotiation, shared problem-solving and recognition of generalists' expertise [72]. A lack of policy and structure to organise generalist palliative care resources in a setting where specialist resources are non-existent often hindered their development [73]. A mentoring model between specialists and generalists could enhance generalists’ palliative care expertise furthering their self-efficacy [74].

Oncologists relied upon family feedback, and positive family feedback was a reinforcement for future referrals. Client feedback helps the clinicians know about a treatment's effectiveness, therapy relationships and adverse outcomes. It alerts them to change the course of treatment and institute risk mitigation strategies [75]. However, in an early bereavement phase, feedback provided by the emotional family members might not always genuinely represent the quality of palliative care services [76].

Oncologists regarded palliative care providers as less competent due to a perceived lack of clarity and objectivity in clinical assessment and management. These findings were corroborated by a study that showed a lack of confidence in paediatric palliative care providers by oncologists hindering palliative care referral [77]. In this study, the oncologists expressed stigma associated with palliative care due to the negative stereotyped association of palliative care with death. Mixed-methods research explored public knowledge, attitudes and perceptions toward palliative care [78]. It showed that general understanding of palliative care is derived mainly from the experiences focused on the end of life care and not the holistic journey contributing to stigma [78].

In this study, oncologists felt that the efforts needed to make a palliative care referral were significant as they had to deal with a palliative care team with limited resources and capacity. Limited access to palliative care was a substantial barrier to paediatric palliative care integration in Eurasian countries [79]. Overcrowding and long waiting times in low-middle-income countries are due to a significantly smaller number of palliative care physicians compared to oncologists, reducing accessibility and effectiveness [80]. The absence of standard referral criteria could make the task of referral daunting. Furthermore, paediatric oncologists are more likely to refer if there is a screening tool for identifying children with palliative care needs and standard referral practices [81].

Oncologists liked palliative care providers to be at the same level as oncologists by introducing them as part of the oncology team. A lack of concurrent care and advance care planning hinders palliative care integration in paediatric oncology [82]. Therefore, these suggestions could fit well with the embedded paediatric palliative oncology model where the paediatric palliative care team is situated in the oncology clinic, ward round and meetings and identified as part of the oncology team [83]. Although embedding is an excellent suggestion for an integrated palliative care model [84], it may not be practical considering paediatric palliative care resource constraints in LMICs [85].

Oncologists liked to rebrand palliative care and introduce it early and covertly as a symptom control team. Oncologists felt that a covert introduction minimises palliative care stigma and family resistance. Negative parental attitudes about palliative care were an essential barrier to referral in a paediatric oncology setting [86]. Children were more open to accepting early palliative care referrals than parents if it offered a symptom control benefit [86]. Literature on early palliative care integration in paediatric cancer settings [69, 87–89] has not explored covert relationships as a strategy for early integration. Perhaps this finding needs further exploration. Oncologists felt training palliative care providers in oncology and paediatrics might improve referral. A lack of palliative care awareness among oncologists [90] and limited access to palliative care providers and resources [79] hindered palliative integration in paediatric oncology. Furthermore, interprofessional palliative care education for paediatric oncologists was identified as a crucial task to facilitate integration [48, 91].

### Discussion of study findings using a critical realist framework

In this study, participants have provided valuable insight into how effective collaboration and a well-developed palliative care team can facilitate referral. Critical realism is a philosophical basis for transformative research [52]. Therefore, these perspectives could bring about emancipatory social change [92] by facilitating palliative care referral and mitigating the pain and suffering of children and their families with cancer.

From a critical realist perspective, there are several generating mechanisms causing the social event; some are empirically observed, and some are deeper and hidden [93]. The interplay of stratified layers of generative mechanisms triggers the event [62]. In this study, the biological layer is formed by illness-related factors such as the stage of cancer, the intent of treatment, cure potential, prognosis, course of illness, and complications. They are the preconditions for a referral. However, biological factors triggering the referral are moderated by a psychological layer. Knowing the referrer's limitations or confidence in their ability, negative sentiments about palliative care providers, trust issues, presuppositions, and past experiences constitute the psychological layer. Social and organisational layers further impact referral behaviour. Family acceptance of palliative care due to the stigma associated with it are examples of the social layer. Availability and access to palliative care, limited resources, hospital-based services, and consultation cost are a few illustrations of an organisational layer.

The study might not be able to fully uncover the stratified ontology of referral [94] and some of the generative mechanisms were not readily apparent [95]. We do not make claims to know or unearth all the generative mechanisms causing referral, and we have explored the perspectives of some participants who can provide reliable and insightful information about the phenomenon [96]. The knowledge generated in this study is contextual to the research country setting[97],, and the perspectives of oncologists about palliative care referral might change over time. It supports the fallibility of the knowledge concept of critical realism, where knowledge about causal mechanisms changes with context and time [62].

From a critical realist perspective, structure, power, generative mechanisms and tendencies are the four concepts that form a perspective [98]. In this study, oncologists discussed various illness-related factors that constitute a structure, which is a precondition for making a referral. Oncologists decide and control palliative care referral, embodying the power to refer by virtue of their status. Their appraisal of benefits and costs of referral, presuppositions, and sentiments about the palliative care providers are some of the tendencies impacting referral behaviour [98]. Therefore, even when structure and power are set in motion, the referral may not be initiated as participants either intentionally or unintentionally choose not to refer [98].

### Limitations and strengths

The palliative care referral phenomenon has oncologists, palliative care providers and patients and families as the stakeholders [99]. The oncologists participating in the research may have different views on palliative care referral compared to palliative care providers, patients, and families. One of the potentials of critical realism is to unpack and understand a complex social phenomenon [100]. This research has unpacked the phenomenon only from the perspectives of oncologists. Future research focused on the views of palliative care providers, and patients and families may facilitate a more comprehensive and holistic understanding of the referral phenomenon.

The research invitation email was sent to several paediatric oncologists across India. Many did not respond, and a few declined the invitation without reason. There is a possibility that participants with a particular view of palliative care responding to the invitation and participating in the study skewed the study results. Only those oncologists who were already referring to palliative care were included in the study as they would have experience with facilitators and barriers to palliative care referral. The views of those who were not accessing palliative care services are unknown. All the participants included in the study are paediatric oncologists working in paediatric cancer units. In India, many adult oncologists care for children with cancer. Their views are not captured in this study.

The research participants were a homogenous group of paediatric oncologists practising in a paediatric cancer unit and referring to palliative care. They were familiar with the phenomenon and provided in-depth, insightful information during the research interviews. The research had good country coverage, and unlike a survey-based data collection, these interviews were generally detailed and provided in-depth information about the research question being explored. However, contextual nature of study findings might limit its generalisability and transferability to other country settings.

### Implications of the study on policy, practice and future research

At an individual level, there is a need for palliative care trainees to have training in both oncology and paediatrics. Likewise, there is a need for oncology and haematology trainees to have palliative care training and to include palliative care in the oncology curriculum. Adult palliative care providers may need additional training in general paediatrics to make them qualified to treat the paediatric population. Besides, a survey study exploring gaps in palliative care education recommended cross-learning between the disciplines [101]. At present, palliative care training is introduced to medical undergraduates in India through a unique attitude, ethics, and communication (AETCOM) curriculum [102].

Research findings suggest that at an organisational level, there is a need for capacity building of paediatric palliative care services. Existing palliative care services could create a separate space for palliative care outpatients to see children and their families. There is also a need to develop community-based services to ensure the child receives care at home and dies at home. There is perhaps a role of federal and state governments to drive this change through strategy, policy, and funding.

At an interdisciplinary team level, oncologists prefer palliative care providers to join them in consultations, family conferences and multi-disciplinary team meetings, which may provide an excellent opportunity for both teams to bond and build confidence. Oncologists preferred palliative care providers to close the referral loop by providing patient information, which could be achieved during multi-disciplinary team meetings. Oncologists have expressed the need for hospital policy and referral criteria. The palliative care team could advocate for an institutional policy on paediatric palliative care that could become a norm for the accreditation of paediatric cancer centres across the country. Creating referral criteria based on disease factors, symptoms and psychosocial needs might advance standardising paediatric palliative care referral. The palliative care community might need to explore a less threatening nomenclature acceptable to oncologists and families to ensure that children in the initial phases of the illness are not excluded from receiving palliative care benefits.

A survey study using a questionnaire developed from these study findings could enhance knowledge of this topic. Moreover, it might be advantageous to know if the adult oncologists’ views on caring for children with cancer on palliative care referral are the same as those of paediatric oncologists. The research participants’ suggestions were for palliative care staff to be part of the oncology team and be present during consultations, family conferences and oncology team meetings. Currently, studies on the effectiveness of embedded paediatric palliative care models are restricted to the United States [103–105]. The feasibility and effectiveness of the embedded palliative care model in the paediatric oncology setting internationally would need further evaluation.

## Conclusion

Competence, capacity building, collaboration, criteria for referral, and changing the name were the key research findings that might facilitate palliative care referral in a paediatric cancer setting. Four themes were inductively generated from the study findings during data analysis. They were: 1) presuppositions about palliative care and palliative care referral, 2) the task of making a palliative care referral, 3) the cost-benefits of making a palliative care referral, and 4) strategies for developing an integrated palliative care model in paediatric oncology. This research had some practice and policy implications. Training in oncology and paediatrics might enhance the competence of current palliative care providers. The capacity to provide palliative care must be augmented by increasing palliative care training positions, hospital and home-based palliative care and increasing inpatient palliative care beds. Close collaboration could be achieved by palliative care providers participating in cancer consultations and meetings. Easy access and completing the communication loop of palliative care referral by providing feedback to oncologists might facilitate care coordination. Clear referral criteria and guidelines on the referral process could clarify referral practices. A change in name to symptom control or supportive care can be explored after seeking palliative care providers’ consensus to facilitate early palliative care integration.

## Supplementary Information

**Additional file 1.** Checklist criteria for good thematic analysis.

**Additional file 2.** Interview topic guide.

**Additional file 3.** Interview transcripts.

**Additional file 4.** NVivo coding process.

**Additional file 5.** Sample coded transcripts.

## Data Availability

All data generated or analysed during this study are included in this published article and its supplementary information files.
