# Supplementary material for: Presuppositions, cost–benefit, collaboration, and competency impacts palliative care referral in paediatric oncology: a qualitative study
Source: BMC Palliat Care. 2022 Dec 2;21:215. doi: 10.1186/s12904-022-01105-0 (PMC9717409; doi:10.1186/s12904-022-01105-0)
Supplement: Supplementary file 1 — Additional file 1. Checklist criteria for good thematic analysis. [file 12904_2022_1105_MOESM1_ESM.docx]

**Checklist Criteria for Good Thematic Analysis**

| Transcription | 1. | The data have been transcribed to an appropriate level of detail, and the transcripts have been checked against the tapes for ‘accuracy. |
| --- | --- | --- |
|  |  | I transcribed the interviews myself, and the exercise of listening to the audiotapes and transcribing enabled accuracy. |
| Coding | 2. | Each data item has been given equal attention in the coding process. |
|  |  | All interview transcripts were uploaded to the NVivo software version 12.6.0 for Mac, and the coding was done using the NVivo software. For each interview, the entire data set was coded. However, only those coded section of the data relevant to the research question was analysed. |
|  | 3. | Themes have not been generated from a few vivid examples (an anecdotal approach) but, instead, the coding process has been thorough, inclusive and comprehensive. |
|  |  | Codes across the datasets were examined for broad patterns, and similar codes (called nodes in NVivo) were collapsed together to create a higher code (top-level node) in NVivo software. A set of these higher codes (top-level nodes) were further collapsed as a candidate theme. The candidate theme represented a central organising concept. |
|  | 4. | All relevant extracts for all each theme have been collated. |
|  |  | Relevant extracts for each theme have been collated through NVivo software |
|  | 5. | Themes have been checked against each other and back to the original data set. |
|  |  | Themes are checked against each other, and themes and subthemes are visually represented in **Figure 5.1.** It is traced back to the original data set using NVivo software**.** |
|  | 6. | Themes are internally coherent, consistent, and distinctive. |
|  |  | Each theme has a set of subthemes that are distinctive and are coherent with the main theme, as demonstrated in chapter five. |
| Analysis | 7. | Data have been analysed rather than just paraphrased or described. |
|  |  | The research findings were analysed and represented as themes/subthemes and interpreted using the social exchange theory and feedback intervention theory. The themes generated were coherent and satisfactorily answered the research findings. Philosophical approach and theoretical frameworks enabled interpretation of research findings |
|  | 8. | Analysis and data match each other – the extracts illustrate the analytic claims. |
|  |  | The analysis of views of oncologists and haematologists on palliative care referral matched the extracts coded and supported the claims made by the researcher. The coded excerpts are provided in the results sections alongside each subtheme in chapter 5. |
|  | 9. | Analysis tells a convincing and well-organised story about the data and topic. |
|  |  | Various aspects relating to the phenomenon of palliative care referral in a paediatric oncology setting and what helps and hinders it was narrated and discussed. |
|  | 10. | A good balance between analytic narrative and illustrative extracts is provided. |
|  |  | Study findings discussed in chapter 5 has a good balance of analytic description of the participant’s views and relevant extracts to support them. |
| Overall | 11. | Enough time has been allocated to complete all phases of the analysis adequately, without rushing a phase or giving it a once-over-lightly. |
|  |  | The data collection and analysis happened over 18 months (July 2018 – Jan 2020). The writing up of the results and their interpretation occurred between April 2020 – March 2021. |
| Written report | 12. | The assumptions about thematic analysis are clearly explicated. |
|  |  | It is stated in detail in Chapter 4 |
|  | 13. | There is a good fit between what you claim you do, and what you show you have done – i.e., described method and reported analysis are consistent. |
|  |  | Themes were organised to describe the phenomenon inductively and reflexively. The subthemes and themes are generated from coded extracts relevant to the phenomenon explored. |
|  | 14. | The language and concepts used in the report are consistent with the epistemological position of the analysis. |
|  |  | Critical paradigm is the philosophical position that informs this research. The research interviews, analysis and interpretation of study findings were consistent with the philosophical position. |
|  | 15. | The researcher is positioned as *active* in the research process; themes do not just ‘emerge’. |
|  |  | The reflexive thematic analysis allows the researcher to play an active role in knowledge creation. The researcher’s subjectivity and reflexive interpretation are part of the data analysis. |
