# Supplementary material for: Presuppositions, cost–benefit, collaboration, and competency impacts palliative care referral in paediatric oncology: a qualitative study
Source: BMC Palliat Care. 2022 Dec 2;21:215. doi: 10.1186/s12904-022-01105-0 (PMC9717409; doi:10.1186/s12904-022-01105-0)
Supplement: Supplementary file 2 — Additional file 2. Interview topic guide. [file 12904_2022_1105_MOESM2_ESM.docx]

Interview Topic Guide

| Sl. No | Questions | Probes |
| --- | --- | --- |
| 1 | Can we please talk about your work that involves managing children with cancer? | Work involving paediatric cancer practice  Experiences of paediatric cancer practice |
| 2 | Can we please talk about the challenges you face during managing a child with advanced cancer? | Child specific challenges  Family specific challenges  Any other challenges?  Why do you think they are challenges? |
| 3 | Can we please talk about your views on the scope or role of palliative care in a child with advanced cancer | Role of palliative care for the child  Role of palliative care for the families  Role of palliative care for the oncologists/hospital |
| 4 | Can you please describe your experience of accessing palliative care services? | How easy or difficult it was to access the palliative care service?  What were the virtues or shortcomings of accessing the palliative care services? |
| 5 | Can you please describe your experience of working with palliative care team? | What were the highlights?  What were the challenges?  How has the experience of working with palliative care service influence your practice? |
| 6 | Can you please discuss your views about referring a child with advanced cancer to palliative care? | What may be the benefits?  What may be the disadvantages?  What would facilitate a referral?  What would hinder a referral? |
| 7 | Can you please describe the situation when you will consider referring a child with advanced cancer to palliative care? | Why? What may be the potential benefits of referral in these conditions? |
| 8 | Can you please describe the situations when you will not consider referring a child with advanced cancer to palliative care? | Why not? What may be the disadvantages of referral in these conditions? |
| 9 | Can you please narrate a memorable experience of making a palliative care referral? | Why was it memorable?  What aspects made it memorable? |
| 10 | Can you please narrate a negative experience of palliative care referral? | Why was it negative?  What aspects made it negative? |
| 11 | Is there anything else you will like to add or discuss that has not been covered in this interview | Probes based on the participant remarks |
