# Supplementary figures and images for: Presuppositions, cost–benefit, collaboration, and competency impacts palliative care referral in paediatric oncology: a qualitative study
Source: BMC Palliat Care. 2022 Dec 2;21:215. doi: 10.1186/s12904-022-01105-0 (PMC9717409; doi:10.1186/s12904-022-01105-0)

Interview Transcripts

|  | 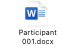 |
| --- | --- |
|  | 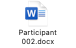 |
|  | 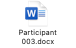 |
|  | 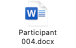 |
|  | 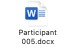 |
|  | 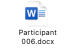 |
|  | 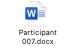 |
|  | 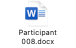 |
|  | 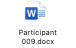 |
|  | 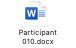 |
|  | 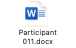 |
|  | 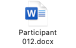 |
|  | 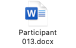 |
|  | 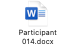 |
|  | 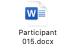 |
|  | 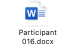 |
|  | 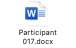 |
|  | 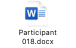 |
|  | 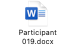 |
|  | 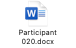 |
|  | 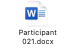 |
|  | 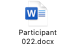 |

Supplement: Supplementary file 3 — Additional file 3. Interview transcripts. [file 12904_2022_1105_MOESM3_ESM.docx]

Coding Process and Organising the Codes using NVivo Software


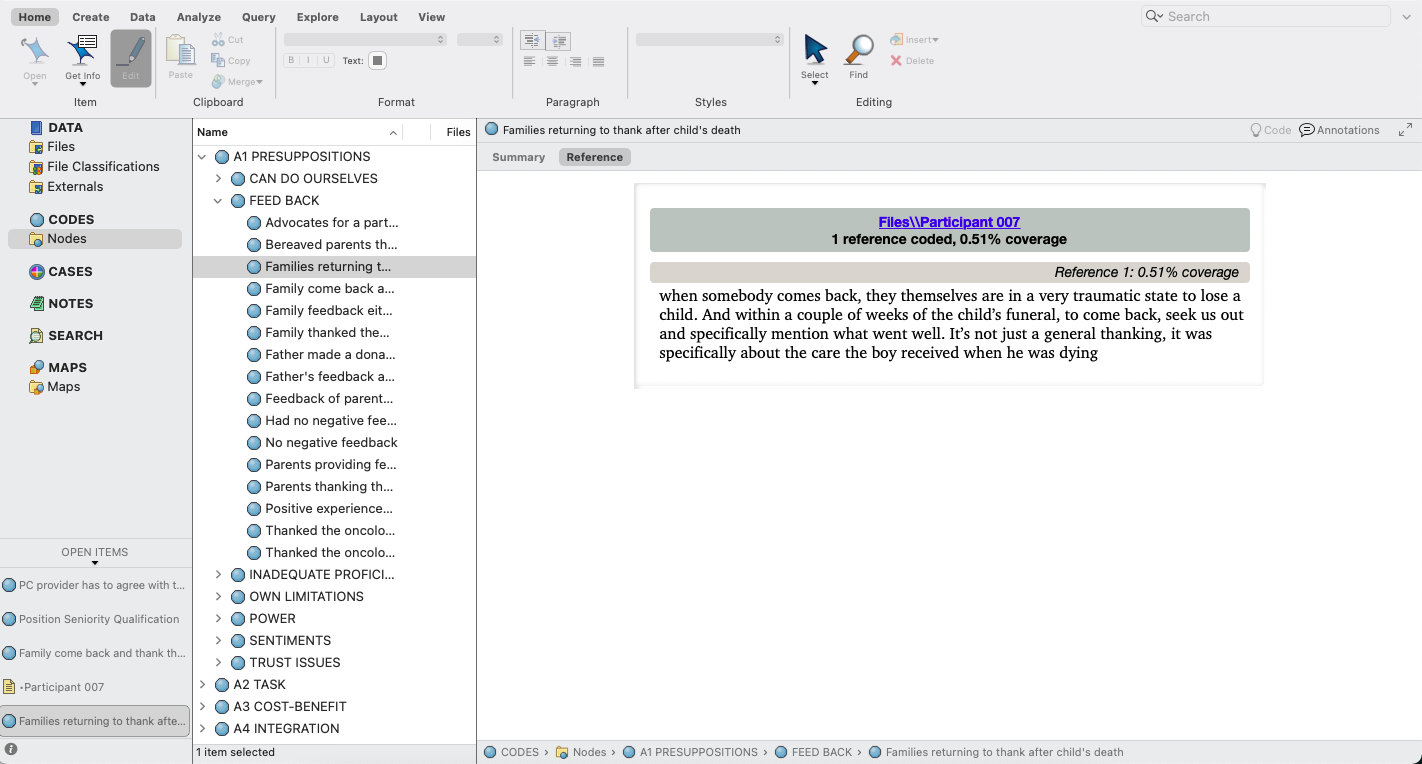

Supplement: Supplementary file 4 — Additional file 4. NVivo coding process. [file 12904_2022_1105_MOESM4_ESM.docx]
