# Supplementary material for: Presuppositions, cost–benefit, collaboration, and competency impacts palliative care referral in paediatric oncology: a qualitative study
Source: BMC Palliat Care. 2022 Dec 2;21:215. doi: 10.1186/s12904-022-01105-0 (PMC9717409; doi:10.1186/s12904-022-01105-0)
Supplement: Supplementary file 5 — Additional file 5. Sample coded transcripts. [file 12904_2022_1105_MOESM5_ESM.docx]

Sample of Entire Coded Interview Transcript

Can we please talk about your work that involves managing children with cancer?

I'm a pediatric oncologist. So, I see children with cancer. Most common is leukemias and the second one is lymphomas and brain tumors. So, I come every day and do the rounds sit in the OPD and go home.

So, what exactly happens when the child comes to you?

When the child comes in for the first time, hmmm, they come with a suspicion of cancer or sometimes they are not expecting it and it's a surprise. (have a suspicion of cancer during the first visit) (surprised as not expecting to have cancer)

So first we do the relevant investigations which is a blood smear or scan or whatever is required and then… (conduct required investigations)

but initially we only tell them that we are suspecting malignancy and we are doing investigations towards that, (at first communicate as confirming the suspicion of malignancy)

so that a warning shot is already fired (smiles) (give an indication)

and they are prepared for the news (to prepare)

and ones we confirm the diagnosis, we will do a biopsy or bone marrow, whatever is required. Once the diagnosis is confirmed then we break the news that it is confirmed as a malignancy and what the treatment is what the prognosis is, what the cure rates are, what the side effects is and then the children start the treatment. (information about diagnosis communicated after thorough investigation) (information about prognosis, treatment and toxicities are communicated before commencing treatment)

You mentioned that they come with not expecting or usually it's a surprise, can we talk about it?

Okay. Sometimes they come with a different complaint where they have not suspected a malignancy. (sometimes present with a complaint not suspicious of malignancy)

And sometimes they are already picked up by somebody and then referred. (sometimes already diagnosed)

In children who are like where they have come with some complaint without a suspicion of malignancy, I think the first thing is once you get the first test which raises your doubt then only we start preparing them like, how this result has come back as abnormal and one of the reasons that is possible is because of this malignancy. (if initial tests points towards malignancy… start preparing the family)

See we need to do the test which would be most of the times…a particular test information, so that they are prepared initially itself that what we are investigating for the malignancy. (inform the family that tests are done to confirm the diagnosis) (helps them to be prepared)

If it doesn't turn out to be a malignancy, they are quite relieved (family is relieved if the tests do not show malignancy)

but most of the times in the suspected malignancy it turns out to be a malignancy. (often it turns out to be malignancy)

Sometimes the answer may be obvious, but we still want a confirmatory diagnosis if the count is very high like two lakh count and you know, there's not much of differential diagnosis is there but still we don't have a confirmatory diagnosis and although we are sure we say that, No, we are suspecting malignancy. We are going to run some tests. That gives them time to digest the information. (even when the diagnosis of malignancy is apparent confirmatory tests are done to prove it) (inform the family that tests are done to confirm the diagnosis) (helps them to be prepared)

Can we please talk about your experience of working in the field of pediatric cancer?

Well, it's actually quite rewarding because unlike adults we cure. (rewarding experience as children with cancer get cured)

Most of the children not all but majority of the children. So, it's much more rewarding doing a pediatric oncology. (most of the children get cured)

And also, you get to know the families because it's a long-drawn process not one consultation. Uh, so you get to know the families you get to know the children and so on. So, it's different from the other specialties. (get to know the families) (prolonged care)

You said it is a long-drawn process. Can you describe more about it?

For families it's always a bad thing, especially if they're coming from different places or where they have to travel. The long-drawn treatment makes it difficult because minimum they will have to stay here for six months or something. There is lot of traveling involved, that way it is bad for the patient. It is good in that you get time to know the family and have knowledge. So, there is much more better understanding between the doctor and the patient. (families have to travel a lot for treatment) (prolonged periods of living at a different place for treatment) (get to know the families) (prolonged care) (bonding between doctor and the patient)

You mentioned that knowing the families has been helpful, can you please elaborate on it further?

Definitely. I think you are at a much more personal level rather than I mean even if it is all professional, even then you know the family, (get to know the families)

you know the names, (know the names)

you know what they are doing and how it is affecting them. (know what they are doing and know how illness is impacting them)

Even where there are short treatment periods when they come for their complaints, you just do not manage the complaint and whatever the disease is. In oncology you get to know the full family and is not just the mother and father, you know, some relatives initially, the people that come and go. So, by the end of the treatment you almost know everyone inside out. (treatment is not limited to managing complaints) (get to know the families)

It is really helpful when you are counseling and the first counselling is difficult then you later know, you know exactly what the issues are. (getting to know families and their issues helps in counseling)

Can we please talk about some challenges that you face in your practice?

Well managing a child with advanced cancer …I think in Indian setting like in any other setting first, the parents are very upset. (parents are very upset with cancer diagnosis)

I think it is even for the completely curable cancers. Once you tell them it's a cancer, they are in shock, denial and all those things… and convincing them… and then there is a phase of like…is there any point in treating… even for curable cancers… (even in curable cancers parents are upset) (react to the information in the form of shock and denial) (family unsure about benefit of treatment)

when you are looking at an advanced cancer with a poor prognosis and all of them becomes exaggerated and not only that they are upset and there is this element of what's the point of treating? (reactions are exaggerated in advanced cancer with poor prognosis) (family unsure about benefit of treatment)

Okay, and then and again the financial and economics comes into play. (family finances influence treatment decision making)

They ask for hundred percent guarantee when we say nothing has got hundred percent here. (family asks for guaranteed outcomes of treatment)

You are telling them it's bad prognosis when percentages are like very low 20% to 30% then it becomes very upsetting for them that they are going to put in a lot of effort, spend and still the prognosis may be bad and they are stuck between whether to do it or not do it. (communication of low treatment outcomes is upsetting for families) (families have to spend and put in effort for treatment) (family unsure about treatment decision making)

I think it is one of the challenges as a doctor you want to do it because even if there is a 10 percent chance you want to try it and see, (doctor would like to treat even when there are low outcomes)

but from the parent’s point of view, even though they came around to that line of thinking initially. There's a lot of uh, which they have to think about whether to continue treatment or not. I think that's one of the biggest challenges. (parents find it challenging to make treatment decisions when outcomes of treatment are poor)

You mentioned parents become very upset. Can we talk about it?

The first thing is to acknowledge that they are upset, and I think showing them hope is okay.

(acknowledge the emotions) (fine to give hope)

That means you have to tell that it is cancer, but there is treatment and it may be curable.

(communicate the diagnosis of cancer also communicate that it is curable)

I think that's like channeling that upset into purposeful energy. So, I tell them, Yes, you have got upset but there's no point sitting upset, you will have to do this. Just let us start the treatment. So, I think that's what takes their mind of upset because they have got something to do. (channeling the upset emotions to positivity by assigning the task of treatment) (having something to do takes their mind away from negative emotions)

You mentioned parents ask you what’s the point of treating. Can you please explain that?

The bottom line is painting the true picture, not very bad, not very good, but to tell them what the percentages are (providing the right information)

what it means because sometimes the most misunderstanding is when you tell the person is 30 percent or something. They think okay. The tumor will come down by 70% and 30% will remain. They don't understand that what I am meaning. So, I clearly telling them what I mean by 30% is like if I treat ten children completely only three will get well. (clear communications to clarify any misunderstandings)

And so that gives some hope that it is not completely lost. So then just let us let us pray and hope that my child is in one of those three. (fostering hope)

Even in the poor prognosis situation it is like all or none phenomenon, some children are completely cured. (even in poor prognosis situation children may be cured)

Okay. So, presenting that data is like explaining to them what prognosis means…not everybody is going to die. (poor prognosis means not everyone is going to die)

There is still hope that a small percentage will live. Yes. It is a small percentage, but there is a chance that your child may be in that small percentage. (small percentage that child may live) (hope that child might belong to the small percentage group)

You have mentioned few family specific challenges are there any child specific challenges that you would like to discuss?

The child specific are…one good thing is children cope much better than adults. (children cope better than adults)

They are not understanding what's happening. (children do not understand what is happening)

Whatever is happening cancer doesn’t mean anything to them. (whatever is happening may not mean anything to children)

It's like they're more worried about the blood test that you are going to do or poking rather than what is going to happen in five years’ time, they don't understand the gravity of the situation. They refuse medications or spit the medications out. (children are more worried about the immediate things happening to them rather than long term outcomes) (refuse medications)

So, there are age specific challenges but overall treating the child is much easier than adults. (treating children is easier than adults)

Less challenges because there is this whole lot of psychological components in adults that are not there. (psychological aspects of care are less in children when compared to adults)

Especially in children less than 5 years, they are just more worried about the immediate problem rather than the overall long-term. (younger children are more worried about the immediate things happening to them rather than long term outcomes)

Challenge is a disadvantage. They don't understand the gravity of the situation of taking medicine or uh, see they take it as like once they are better than there's no need to take any medicines. (children not able to understand the seriousness of illness can be challenging)

It is quite upsetting for them that they don't know why it is being done. Once they improve a little bit that they are actually well and feel that they are completely well. So is more difficult for them to accept that they are well, but something is being done to them. There's no explanation. Like we are saying we are trying to make you better. Like I'm already better isn't it. (children find it difficult to accept treatment not knowing why it is done and done when they are well)

In other situation like respiratory distress, they accept a little bit better because they are having a problem that is getting improved. And once the Improvement happens, whatever hurt is being done will stop. In oncology they come with some problems, symptoms improve and then they're actually well, but you're continuing the treatment. (children accept treatment when they are unwell) (children find it difficult to accept treatment when they are well)

The other extreme is the teenager. In the teenager because problem is again complex. (treating teenager is complicated)

Okay, uh, because the ones that are well, they understand everything, but do not want to do. (although teenagers understand they refuse)

The problem is once they have a hair loss then they don't want to go to school. They don't want to go to because the other children will make fun of them. (do not want to go to school when they lose hair) (fear ridicule from their peers)

Even if it doesn't happen, they are afraid that that is going to happen. Even though they are fit to go to school. They don't go to school. (fear of losing hair prevents from going to school)

There is an element of overprotection from the parents also that is adding to not going to school. I clearly tell them to send the child to school now but still the child is kept at home.

(parental overprotection prevents children going to school)

It is because one the parents overprotect, and the other thing is the child doesn't want to go because there is no hair or there is line sticking out. (parents do not want child to be sent to school when there is an IV line)

This is especially in the school going children. In the older children like about 15 to 16, the main problem is the compliance. Um, they don't think I will get better. I don't like, oh I got cancer. I don't want to do anything. I don't care attitude. (compliance and attitudes of teenager towards cancer and treatment can be challenging)

Are there any other challenges apart from what you have mentioned that you would like to discuss?

I think the main problem…. Actually, in the Indian setting it is the finances it is the economic. (financial issues pose the major challenge)

Firstly, the treatment is costly, and it is not one of off treatment. (cost of treatment and prolonged treatment)

So, it is emotionally draining and physically draining for the family. (emotional and physical drain)

The other thing is the uncertainty, one complication can happen as everything goes well, so you tell them the plan and this one complication will throw the entire plan out of the window. (uncertainties due to complications)

I think that's where the families find it very difficult to cope. (families find it difficult to cope)

A child comes in with like leukemia something then one severe infection, they are in the ICU, uh, not only the condition changes, the cost planning that they have planned goes for a toss. (development of a complication during treatment upsets the treatment cost planning)

You mentioned few financial challenges, can you please discuss it further?

I know but there's one thing good thing about this we can try to cut the costs. (treatment costs should be reduced)

Hospital will provide discounted treatment and the other way now is the crowdsourcing. I think the lot of patients are able to go on the crowdsourcing website and are able to raise funds for the treatment. (discounting treatment and crowd funding)

There are certain things that we can do where we can cut the antibiotics orally. Things like that can be done. And the other thing is the hospital giving some discounts and like that so that is how I think the financial parts are being managed. (minimizing antibiotic use) (hospital discounts) (managing finances)

**Can you please describe your experience of referring or accessing palliative care?**

I think I mean, I worked at setups where there was established palliative care. Even when I was in the setup where there was no palliative care in-house, there are always access to a palliative care, a good palliative care team. (worked with an established palliative care setup) (had access to a good palliative care team)

So, I don't think there was any major hurdles in accessing the palliative care. (no major hurdles to palliative care access)

When we are talking about access, I would like to know how easy or difficult it was to refer to palliative care?

I think it was quite easy because well, when I was in ****** there was a good palliative care team in place. (had easy access to a good palliative care team)

It was easy to refer and when I moved to the next hospital, there was no in-house palliative care team.

(hospital palliative care team makes it easy to refer)

So, most of our advice was through the phone, but when the patient was discharged, and I handed over to the palliative care team. (received phone advice) (care transferred on discharge)

I see the palliative care team as like two things one is inpatient for immediate symptoms for the admitted patients and the second part is once I sent them home for ongoing care. (role of the palliative care team perceived as: symptom control and ongoing care after discharge)

In places where that is inhouse palliative care team, both parts are taken care of well.

(both the roles performed well by the hospital palliative care team)

Okay, palliative care teams will assess the child and put their plans, (assess and plan treatment)

symptom relief and everything else. (symptom control)

And then even after discharged home the palliative care takes over. (care at home transferred to palliative care on discharge)

Whereas working in a setup where there is no in-house palliative care team, Then the problem becomes when the patient is admitted under me (patients admitted under the oncologists when there is no hospital palliative care team)

and I am just taking palliative care advice on phone. (receives phone advice)

I just tell this the symptoms and everything that the quality becomes poor, a little bit because obviously, (quality of palliative care treatment compromised)

uh, I won't be able to do as good an assessment as a palliative care team would do from that point of view. (inability to conduct a good palliative care assessment)

But it still helps when I present them. They will tell something. (receives palliative care advise based on oncologist reporting)

Once discharged I just hand over completely to the palliative care team. (care transferred on discharge)

You just mentioned good palliative care team. Can you please elaborate what “good” means?

I said good. Good means proactive. (proactive care)

Not just I mean once you refer a patient, uh, you would come see the patient assess the patient. (patients are assessed by the palliative care provider on referral)

It is not like you give a problem, like pain, you come and give Morphine, not that kind of palliative care, but actually assess, check, look at all the aspects. Okay, and then come up with a full plan rather than telling patient has pain give Morphine. (not a problem check-list based approach) (assess all aspects of care)

It is quite easy to tell; we are not asking for the dose of Morphine. What you're looking for is the complete approach and package which is there in the palliative care. (expecting beyond problem check-list based approach)

Can we please discuss about the highlights or challenges in making a palliative care referral?

They were always at the end of a Phone call, (always available on phone)

uh, maybe I uh, one thing was the only one thing was out of hours availability. (limited out of hours availability)

Otherwise within one working day all the patients used to be seen. (patients seen within a day)

Once you give a referral they will see on the same day or whatever the phone advice was given and by the next working day the patient was seen. (patients seen the same day or the next day)

At ***** actually as far as accessing the palliative services or getting hold of a palliative person was not difficult in the last few years. (accessing palliative care services was not difficult)

Can we please discuss about your experience of working with the palliative care?

When I returned from UK, I thought that there is no palliative care in India, (expected that palliative care was unavailable in India)

which was true to a certain extent, but I was just lucky (chance)

that I have to land in a place where there was a palliative care team in place. (joined a place where palliative care was available)

90% of the cases where I could have landed there may not have been a palliative care team in place. (high chance of joining a place where a palliative care team was not available)

I was just lucky enough to come to a place where there was an ongoing palliative care team, (considers himself lucky)

but I think most of the places they don't have access to palliative care team and there is lot of deficit. (feels that there are gaps in access to palliative care in most places)

My experience was very good (good experience)

to the extent now I just spread the word that this person is good, you can refer to him. (advocates for a particular provider)

For many other they don't even have it, (majority do not have palliative care services)

I mean they might have just heard about palliative care or it is something which they have read in the textbook, they have not actually accessed it. (majority have knowledge about palliative care from books not from experience)

I put lot of patients in touch with Dr. *****. You can refer to this person. He does a good job. (advocates for a particular provider)

Can you please narrate some of the highlights of your experience?

Parents of some of the children who died actually came and thanked us which is very unusual.

(parents thanking after the child’s death is unusual)

When the child dies in the acute setting the parents are very upset,

(child’s death in an acute setting is upsetting)

whereas children who have been referred to palliative care, some parents actually come and thank us, my child died, but still we thank you. (when referred to palliative care, bereaved parents thanked the referring oncologist)

You tried your best and the child was not in pain and was comfortable. And some of the patients were actually kept comfortable at home. (child was pain free, comfortable and at home)

I think that is one of the things which was really highlight as all these patients didn't die in the hospital. They died at home and (dying at home was the highlight of the experience)

I'm sure most of the palliative care teams would have been giving advice on the phone to these families. (providing phone advice to families)

Once I handover, I just roughly know what's happening but not actually involved in the actual care of what's happening? (knows somewhat about what’s happening with the child even when actually not involved)

I know this is going this is happening and the child is comfortable. (knows that child is comfortable)

I get to know the information, but I am not like running the show of increasing decreasing the doses or responding to the phone calls. (not involved in the day today care of the child)

And so, whenever I handed over a patient to a palliative care, then I get an occasional phone call about the patient. (receives information through occasional phone call)

Most of the patients whether admitted or the treatment has started in the hospital or in the OPD. These patients also actually died at home (hospitalized patients referred to palliative care died at home)

and I am sure the palliative care consultant would have seen patient only once or twice physically, (patient seen only a couple of times by the palliative care consultant)

but the whole care was continued at home (care continued at home)

I think that was one of the good things about working with the palliative care team. (team approach)

You mentioned that it is good for children with terminal cancer to die at home. Can you explain it further?

I think it depends on the family. Some families can't cope. Okay. Some families just cannot cope. (some families find it difficult to cope)

They want to bring the child to the hospital and let the acute service take care when the child dies. (families want the child to die in the hospital)

Even though there is no point in treating they come to the hospital because they do not want it to happen at home. (families do not want the child to die at home)

They are actually afraid of what is going to happen when the child dies. (families scared of the events surrounding death)

They have accepted the fact that child will die (accepted child’s death)

but the actually, what is going to practically happen they don't know (not aware of the events surrounding death)

so they bring the child to the hospital to die. (child brought to the hospital to die)

Where the palliative care team was involved and like there was time to develop rapport between the palliative care team and the family, none of them came back. (rapport with the palliative care team) (child did not return to the hospital)

They all accepted, knew what was going to happen and those children died at home. (accepted child’s death) (knew the events surrounding death) (children died at home)

So, in a way that was good, but I think that depends… what decides that is one the family, and more than family how much time there was between the referral and the death. So, like where there were more than 15 days or one month or something a rapport actually developed between the family and the palliative care team. (early referral facilitated building rapport between families and palliative care team)

They have been much more comfortable to keep the child at home. (family comfortable with keeping the child at home because of previously developed rapport)

You just mentioned about duration of palliative care input and outcomes. Can you please elaborate it further?

I think it is very important and when I was in **** it used to be introduced as symptom relief team and patients knew the palliative care team at diagnosis. (palliative care introduced as symptom relief service) (patients were referred at diagnosis)

It was not just the palliative care team, it was a palliative care team, but they were doing symptom relief and presented as yeah, we treat the cancer and these people treat the symptoms. So, the family already knew them. (oncologists treat cancer palliative care providers provide symptom relief)

It was not like from today you are palliative. There was no point where uh today you are palliative care… kind of switch over, it was continuous. (not switch over but continuous)

While working at ****that switch had to be done because there was no inhouse palliative care team to involve. (switch over happens when there is no hospital palliative care team)

So, the symptom control was being done by me only and it used to be like, (oncologist has to perform the symptom control role)

okay you are now palliative; this person will take more precedence over what I am doing. (switch over to palliative care)

You spoke about transition and rapport building; can you please elaborate on it?

It is a clear-cut transition here; there it was smooth. Actually, there was no transition at all. The palliative care continued to look after the symptoms, and we used to say now we will start treatment to cure your child. It was not like this is on that is off. It was like both is on and later one is off. (oncologists and palliative care providers performing their roles concurrently) (no transition issues when palliative care providers are working concurrently with the oncologists)

I was lucky enough to work with good people. (worked with good providers)

The only challenge was when I moved into the hospital that did not have palliative care. (changed jobs and had to work in a health care set up without access to palliative care)

I was so used to palliative care, which was looking after everything. (used to working in a set up with access to palliative care)

When I was working at **** that had an in-house palliative care, I did not have any challenges. At the other place it was the access to palliative care which was not there. (lack of access to palliative care was challenging)

You have stressed upon the need for an inhouse palliative care. Can you explain it further?

I think there is a need for inhouse palliative care. Yes, especially if you're dealing with uh, life limiting conditions. (hospitals caring for people with life limiting illness should have a hospital palliative care team)

In my previous workplace, it was the children’s hospital and I was the only person treating cancer, the others were like respiratory physicians treating cystic fibrosis. People felt that there was no need for a palliative care team. (palliative care need is felt less among non-cancer providers in a general hospital)

When I was working at ****, it was a cancer hospital, so it was available. (palliative care need is better felt in a cancer hospital)

When you are working in a general children's hospital, very few people access the palliative care team. (fewer providers access palliative care in a general hospital)

Can we please discuss your views about referring a child with advanced cancer to palliative care?

I think when you are going down advanced cancer prognosis diseases…I think the quality of life becomes important. (quality of life is important in advanced cancer)

Okay, so, whatever we do. I think the palliative care team has got a better approach in providing the quality of life than an acute physician. (palliative care providers stand a better chance in improving quality of life than acute care providers)

I think it is important to refer to palliative care. (referral to palliative care is important)

You mentioned about few benefits, can we talk more about it?

I think benefits, one is child is more comfortable. (child is made comfortable)

Better symptom control (symptoms better controlled)

and number two there is less use of the hospital. (less access to hospital resources)

Okay, because just by training we are more used to see the patient, take history, do the clinical examination and then treat. When you are an acute physician you still treat the palliative patient with the same approach. (acute care physicians continue to treat a patient needing palliative care with acute care approach)

So, you are uncomfortable telling something on the phone without seeing the person. (acute care providers are uncomfortable in giving phone advice)

The palliative care person is much more comfortable on phone and managing at home doing that. (palliative care providers are comfortable in giving phone advice)

Okay, so actually place like**** where patients have to travel to see you, I feel that why they have to bring the child to the hospital for such a small complaint, but you are not confident enough to manage the child at home because your training is like that. Because you don’t what to miss something. In acute medicine, you want to do a good physical examination, then you approach the problem. (by training acute care providers insist on bringing the child to the hospital for assessment) (bringing the child every time to the hospital for minor complaints can be an inconvenience)

And you even see the palliative care from that angle because that’s what you're doing every day. The palliative care physician is comfortable answering over the phone and keeping the child at home. (palliative care providers are comfortable in giving phone advice and managing the care at home)

You mentioned about how palliative care is important to improve quality of life of the child. Can you please explain it further?

I think one is symptom relief. Okay, uh, no pain, no vomiting and wound care, (relief of symptoms)

Okay, and actually managing the patient at home, (home based care)

that actually will improve the quality of life that they are not carrying the child in **** where it will take half a day to come and go to the hospital. That itself improves the life. (logistics of travel and time taken to access hospital)

I'll tell you an example. I had a child with rhabdomyosarcoma, which was recurrent. And it's spread to the brain and all I referred the child to ****. Because this was admitted and operated in **** then discharged and palliative care was not there. Then after the surgery I did not see the patient at all because the child was too sick and bedridden to bring to the hospital. ****just saw the child once and I think he managed the palliative care for like three to four months and actually when the child died, they phoned me up and thanked me. (child referred to palliative care died at home) (family called the oncologist and thanked)

I thought **** was like actively seeing the child and managing the symptoms, but later I realized that **** has seen this patient once in the OPD. (child was seen at palliative care outpatients only once)

And everything was managed on the phone (phone advice and care was managed remotely)

and the family was really grateful because the child was bedridden, and it was not a very small child to carry. To bring them to the hospital even for symptom control would have been very difficult. (family expressed gratitude as it saved them the logistic challenges of transporting the child to the hospital)

I think that’s the quality of life and that's like a real improvement in quality of life. If not anything else… not making them travel. (minimizing logistic challenges of travel and transporting the child back to the hospital is a way of improving quality of life)

Can we talk about any disadvantages of palliative care referral?

The only disadvantage I can see is the naming (smiles). (the name palliative care is disadvantageous)

The Palliative, because the patient feels that you are already in the last stage. (patient feels palliative care means end stage)

Okay, so that's why it's better to get them as symptom control and introduce early. (introduce the service as symptom control and introduce early)

You talked about naming or branding somebody as palliative. Can you please explain it?

It has both advantage and disadvantages. When you brand as palliative then the realization happens to the parents. (naming someone as palliative can help the parents to realise)

Okay. This is now we are on a different territory, we are not trying to cure, that kind of realization it is good. (can help them to realise the change in treatment approach) (realisation is good)

But when parents have not accepted. Okay that there are some chances of cure etc. there will be resistance to go to palliative care. (parental resistance to accept palliative care when there are some chances of cure)

But that can be overcome by telling that this is for symptom relief. (introducing it as a symptom relief service helps in overcoming the barrier)

It does not matter that I am trying to cure you or keep you comfortable, keeping you comfortable is important. (child’s comfort is important irrespective of treatment approach)

Even If I am trying to cure you, you need to see palliative care (palliative care is needed during the curative phase)

Not just rebranding, I think it should be from the beginning, right when you write the first dose of Tramadol or Morphine, the palliative care should be involved. (palliative care should be introduced early)

The patient sees you as not as palliative care, not like a switch on and off but a continuous service right from beginning for the symptom relief. This is the team which will come and give the relief. I think it is the introduction at the first stage rather than the last stage. (continued service from the beginning for the relief of symptoms)

You mentioned about certain challenges because of the term palliative care, can we talk more about it?

At this point no. Once I moved to the**** it was always a switch on and off kind of thing. (switching over of the services)

It is because they understood that we cannot cure he's handing over to a person who will do the symptom control better. (when curative options are exhausted transfer to the provider who can control symptoms)

Can we please discuss what facilitates or may facilitate a palliative care referral?

I think in the India setting availability. Availability (availability of the services)

and knowing the right person. (right provider)

Even now I think that the service is patchy and not all hospitals have access to palliative care. (access to palliative care is limited)

You mention about the right person, can you please discuss what do you mean by that?

Because in settings where there is no actual palliative care team the job is taken over by a person who can manage pain, usually the anesthetist. (when there are no palliative care services pain physician and anesthetist fills in the role of the palliative care provider)

Okay, and their approach is entirely different. (pain physician and anesthetist have a different approach)

Okay… The anesthetist comes in because there is lack of service, you know, you want to refer to palliative care service but there is no palliative care service and what is the next best option? The next best option is the pain team which is usually run by the anesthetist.

(anesthetists and pain physicians are the next best option when there are no palliative care providers)

They get the control of the pain. very good, they do their job very well, but it doesn't cover the other things. (role limited to management of pain) (other aspects of palliative care are lacking)

When you say other things, what do you have in your mind?

When you say other things, I mean the not only the symptoms, but taking on social, emotional and psychological. Things that are completely untouched. (palliative care is not limited to managing physical symptoms) (Involves other health dimensions) (Other health dimensions not addressed)

Can we please discuss what hinders or may hinder a palliative care referral?

As far as I can see the main hindrance is only the availability. (non-availability of the service hinders)

Availability of the right person in the hospital. (right provider)

Once I decide, when I say an advanced cancer, the first thing I do is to counsel the family. (counseling the family about advanced cancer)

This is advanced cancer. These are the chances. Do you want to take the treatment or not take the treatment? These things are mentioned. (counseling about outcomes of the cancer and treatment decision making)

Most of them they will try to take treatment (majority opt for treatment in advanced cancer setting)

and once it fails, at that point the child is still asymptomatic. Okay, because we are going to pick it up early on the scan or a blood result or something, rather than the child being acutely symptomatic. (children with advanced cancer are often asymptomatic) (advanced cancer often picked up on investigations)

But you know, which way it is going to go. I usually refer to the palliative care team at that point before the child has got any symptoms. (refers the child to palliative care before appearance of symptoms)

When I say okay, this is now the time the child who is going to go from trying to cure to trying to keep the child comfortable. (discusses shifts in goal of treatment from cure to comfort)

Right now, completely comfortable but that is the time I introduce. I tell them I know your child has no symptoms now. Your child is likely to develop symptoms later. (asymptomatic now may develop symptoms later)

You will need the help of this physician, please go ahead and see him now. (referred before symptoms appear)

You mentioned referral should be made before the child develops symptoms. Can you please discuss it further?

As I told you, first the rapport should develop. So that they know the physician. (referring early before symptoms develops builds rapport)

I think then it's much easier for the family also, to access palliative care, rather than wait for the symptoms to develop. (referring early when symptoms have not developed allows time for accessing the services)

All my references have been at that point, where I know that the child is palliative, but the child is fine. (always referred when there was a change in the course of illness)

So, they still go and see. He may say nothing, and everything is okay, if there are symptoms call me. (palliative care consultation in absence of symptoms may not yield much but important for future care)

You mentioned about referral when the treatment fails. What do mean by treatment failure?

The example, I was giving earlier, when I discharged, the child was fine. But it has recurred, and the surgeon told he cannot operate on it. That is the only time **** saw the child. There was no symptom actually and the child was well, okay. I'm not going to try to cure this child. There is no more aggressive chemotherapy radiotherapy or surgery. (child had advanced illness with no curative options) (child was asymptomatic)

We are not going to go down that route from now onwards and I know that this child is going to die but let me keep this child as comfortable as possible for as long as possible. (change in the treatment course, poor prognosis and the goal was comfort)

Are there any situations in your practice where you will refer the child to palliative care more often than other?

I think mostly, more often solid tumors tend to get referred than leukemias, although leukemias form the biggest number. (solid tumors are more often referred compared to leukemias)

I think that is because leukemia relapses are much more acute, and the child is going to die because of the low platelet count or something because of which the child is going to bleed. The time interval between stopping the treatment and the child actually dying is much shorter in the leukemia (acute course and complications in children with leukemia makes palliative care referral challenging)

compared to a solid so by default the solid tumors tend to get more referred that too extra cranial. (extracranial solid tumors are more often referred compared to brain tumors)

Because with the brain tumors what's happening most of the times is that the child gets seizures, unconscious or has drowsiness and all uh the patient brought to the hospital just because of the nature of the illness. (acute course and complications in brain tumors makes palliative care referral challenging)

Child having a solid tumor having pain or vomiting or other symptoms are more accepting of the palliative care physicians’ approach. (symptomatic children accept palliative care more easily)

But in leukemia it is very clear, they will need platelet transfusion or blood transfusion and by default end up in the hospital. The same thing is for the CNS tumors, because it will be a fit or the child becomes drowsy. Very rarely they may have hemiplegia and palliative care physician will be looking after them. But most of the time the child starts fitting, becomes drowsy, sleepy, abnormal breathing then they tend to bring the child to the hospital. (leukemias and brain tumors present with complications and acute care needs needing hospitalization and care from an acute care provider)

So, by default more solid tumors end up with the you. (patients with solid tumors are more often seen by the palliative care provider)

It is because I think it's a more long-drawn process. (protracted course of cancer favors palliative care)

Because if the child is fitting or coning, or the child has a big intracranial bleed, or pulmonary bleed, we are not going to intubate, and the child is going to die very quickly. (cancers with acute complications can have a short course before death)

In solid tumors it is a long-drawn affair. So, there is more period which the child needs care (protracted course of illness in solid tumors favors palliative care)

Are there situation when you have not considered referring a child with advanced cancer to palliative care?

So far, I have not thought of any. I think it has to do with the acceptance of the family, (family acceptance of palliative care key for referral)

I think if you have not referred because the family is in denial and don’t want to see palliative care. (non-referral is due to non-acceptance of palliative care by family or denial)

Very rarely almost none that I have not offered. (seldom not referred)

If I see this as an advanced cancer with symptom needs which is beyond my first line of management I usually refer. (advanced cancer, symptoms, beyond first line of treatment triggers for referral)

I usually tell that this is a person who can take care of this problem better than me then it's up to the family to accept or reject. (decision to accept/reject rests with the family)

I don't use the word palliative care. I just say these kinds of symptom is looked after by this person. He will do a better job. (name palliative care not used) (introduced as symptom control service)

You mentioned about the situation where palliative care is required but family is refusing, can you explain it further?

They are a bigger problem because they tend to end up more landing in the hospital. (families refusing palliative care more often access hospital resources)

That is why they just bring the child to the hospital and stay there till the child dies. (families refusing palliative care have higher chances of child dying in the hospital)

Then everything is taken control off, you put the canula, just keep going up on the painkillers. You control everything, everything is taken care of. (receive more intense acute care in the hospital)

It is exactly the type of parents who are not able to cope with the process of child dying at home. So, they will line up in the hospital, just come to die. (unable to cope with child dying at home) (accessing hospital resources and hospital death)

And you will have to keep the junior doctors away from acting on things. (have to resist junior doctors from acting upon things happening with the patient during the course of hospital stay)

You mentioned acting on things, what do you mean by that?

When I say acting on things, because some will ask for the blood sugar to be checked or CBC, where it is completely not required, and the child will be poked three times to take the blood which we are not going to act. (medical investigations not required, or no action will be taken on the investigation results)

Can you narrate a memorable experience of palliative care referral?

I've told you one that we have managed. This was a child who had a tumor in the paranasal sinuses operated. Given chemotherapy and radiotherapy, recurred, given second line, again tumor regressed, and then was sent for surgery, the second time surgery went wrong because they couldn't remove the entire thing. So, they did half as much excision as possible and came out and then they went in again to remove that tumor (child with a recurrent advanced cancer)

and the child had complications, intracranial bleed, hemiplegia and all those things. So, they abandoned the surgery then they wanted to operate on the child again. (severe complications)

But by that time, she was not fit for chemotherapy. (not suitable for disease modifying treatment)

She was waiting for the surgery and the disease progressed. Progressed means only on the scans. (progressive disease)

The child had no symptoms and that is when we decided that the child is progressing on second line chemotherapy. (progressed on treatment)

They went and saw ****. Finally, the patient died, the parents actually phoned and said I'm so sorry the child died, but you did everything and **** did everything and thanked us. (family called and thanked the oncologist after patient’s death)

Why was this episode a memorable experience?

The fact that the child was managed at home without ever getting out of the home was like really unique. (continued care at home until the end)

Can you narrate a negative experience of palliative care referral?

As far as I can see, the team that I have worked with, I think no.

Is there anything else that we have not covered in the interview you would like to add?

The only thing I would like to add is more of you are required. Availability is the main issue. (availability of the services is the main issue)

Since this question of availability had come time and again why do you think palliative care services are not available to paediatric oncologists?

I think right now because there are more pressing needs. Okay. Now the biggest challenge is to actually convince the patients to get to take the treatment and not to abandon the treatment. Most of us now are focusing our efforts towards that. Until this issue is sorted nobody is going to ask for more palliative care. All the efforts are towards curing the children who can be cured. We are failing in that itself. That is seen as a bigger need. (not taking treatment or abandoning cancer treatment is a major challenge) (efforts focused towards enhancing cancer treatment takes precedence over palliative care)
